# Discovery of Novel µ-Opioid Receptor Inverse Agonist from a Combinatorial Library of Tetrapeptides through Structure-Based Virtual Screening

**DOI:** 10.3390/molecules24213872

**Published:** 2019-10-27

**Authors:** Giulio Poli, Marilisa Pia Dimmito, Adriano Mollica, Gokhan Zengin, Sandor Benyhe, Ferenc Zador, Azzurra Stefanucci

**Affiliations:** 1 Department of Pharmacy, University of Pisa, Via Bonanno 6, 56126 Pisa, Italy; giulio.poli@unipi.it; 2 Department of Pharmacy, University of Chieti-Pescara “G. d’Annunzio”, Via dei Vestini 31, 66100 Chieti, Italy; marilisa.dimmito@unich.it; 3 Department of Biology, Science Faculty, Selcuk University, 42130 Konya, Turkey; gokhanzengin@selcuk.edu.tr; 4 Institute of Biochemistry, Biological Research Center, Hungarian Academy of Sciences, H-6726 Szeged, Temesvári krt. 62, 6726, Szeged, Hungary; benyhe.sandor@brc.hu (S.B.); zador.ferenc@brc.hu (F.Z.)

**Keywords:** peptides, virtual screening, μ-opioid receptor, pharmacophore, docking

## Abstract

Morphine, oxycodone, fentanyl, and other µ-opioid receptors (MOR) agonists have been used for decades in antinociceptive therapies. However, these drugs are associated with numerous side effects, such as euphoria, addiction, respiratory depression, and adverse gastrointestinal reactions, thus, circumventing these drawbacks is of extensive importance. With the aim of identifying novel peptide ligands endowed with MOR inhibitory activity, we developed a virtual screening protocol, including receptor-based pharmacophore screening, docking studies, and molecular dynamics simulations, which was used to filter an in-house built virtual library of tetrapeptide ligands. The three top-scored compounds were synthesized and subjected to biological evaluation, revealing the identity of a hit compound (peptide 1) endowed with appreciable MOR inverse agonist effect and selectivity over δ-opioid receptors. These results confirmed the reliability of our computational approach and provided a promising starting point for the development of new potent MOR modulators.

## 1. Introduction

The new era of opioid studies began with the isolation of endogenous ligands in the brain for analgesic receptors, which led in a short time to structure determination of enkephalins, dynorphins, and endomorphins, as well as the opioid receptors µ (MOR), δ (DOR), and k (KOR). The putative physiological roles of opioid peptides and their associated receptors have been under intensive investigation for many years and, in the beginning, the cloning of opioid receptors has provided direct structural evidence of “multiple opioid receptors” [1,2]. Although the cloned opiate receptors represent powerful tools for the physiological and pharmacological evaluation of their roles in normal and painful states, very few new insights have been made to discover ligands capable of maximizing efficacy, especially in vivo, against neuropathic pain. Opiate therapy is one of the most commonly prescribed treatments for chronic neuropathic pain; however, opioids do not address the mechanisms of neuropathic pain and often have limited efficacy against this type of pain [3]. Opioids are plagued by analgesic tolerance, addiction, medication overuse, hypersensitivity, and other physical side effects. In addition to social and legal issues associated with their use for non-medical and recreational purposes, several adverse effects (e.g., dysphoria, constipation, respiratory depression, nausea, vomiting, etc.), hinder their clinical usefulness and justify the discovery of safer opioid therapeutics and/or non-addictive medications [4].

**Figure 1:**
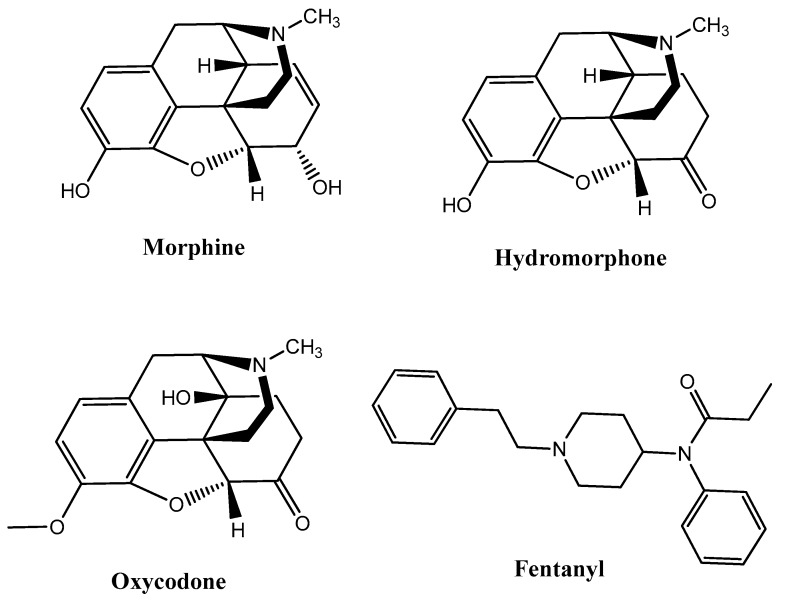
Most of the representative µ-opioid receptor (MOR) agonists reported so far.

G protein-coupled receptors (GPCRs) are characterized by a basal signaling activity in the absence of agonists in over-expressed receptor cell lines and at physiological receptor levels [5]. Compounds acting as antagonists can block agonist-mediated receptor activation but may exert different effects at basally active receptors, thus suppressing basal signaling activity as inverse agonists (antagonists with negative intrinsic activity) or neutral antagonists. However, there are also antagonists that don’t affect basal signaling. Dopamine D_1_, D_2_, and D_3_ receptors, β_2_-adrenergic receptors, adenosine receptors, serotonin (5HT_2A_) receptors, and δ-opioid receptors (DOR) are able to bind inverse agonists and neutral antagonists [6]. Intrinsic efficacy depends on the context of the cells, receptors, and experimental conditions. Information about the physiological role of basal signaling activity of receptors and the therapeutic importance of inverse agonists or neutral antagonists are still scarce and uncertain. For instance, diverse intrinsic activities of β-adrenergic receptor blockers influence cardiac contractility and possibly the therapeutic outcome of patients with cardiac failure [7]; moreover, the high constitutive activity of native H_3_ receptors regulates histamine neurons in the brain [8]. Considering the therapeutic perspective, MOR agonists such as morphine, hydromorphone, oxycodone, and fentanyl discovered through traditional approaches, such as natural product isolation (e.g., morphine), semi-synthetic natural product derivatives (e.g., oxycodone, hydromorphone), and synthetic manipulation of natural product scaffolds (e.g., fentanyl), have been used for decades in antinociceptive therapies (Figure 1).

These are potent μ-opioid agonists that exert addiction liability and numerous side effects, such as euphoria, addiction, respiratory depression, and gastrointestinal adverse reactions; therefore; circumventing these drawbacks is of extensive importance. A large amount of literature documented novel approaches to disconnecting the analgesic efficacy of μ-opioid agonists from morphine-like side effects, for example biasing the GPCRs over β-arrestin2 recruitment (TRV130, PZM21, HS665) or designing positive allosteric modulators of the MOR (BMS-986122), MOR inverse agonists/KOR antagonists [9], and multiple agonists of opioid receptors subtypes (SNC80, DPI-125) [10]. Different peptides and peptidomimetics have been reported in the literature to exert a strong antinociceptive effect on opioid receptors without involving the β-arrestin protein, which is thought to be responsible for respiratory depression and constipation associated with the use of naturally occurring and synthetic opioids. The natural peptides rubiscolin-5 and -6 are excellent examples of molecules that activate G protein signaling pathways at the δ-opioid receptors, with minimal β-arrestin recruitment [11]. Thus, nowadays the design of small molecules with a peptidic structure could be a valuable strategy to achieve selective activation of a desired opioid signaling pathway, reducing the incidence of unwanted side effects.

**Figure 2:**
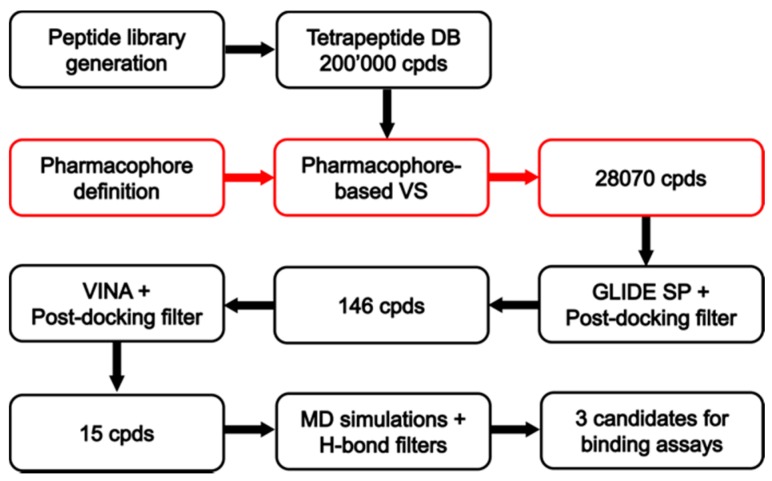
Schematic workflow of the receptor-based virtual screening (VS).

Receptor-based drug design is a precious tool to furnish a detailed molecular-level understanding of the interactions between opioids and their receptors. High-resolution crystal structures of all four opioid receptor subtypes, i.e., the MOR, DOR, KOR, and nociceptin/orphanin FQ receptors, allowed for the development of virtual screening (VS) campaigns that led to the discovery of novel chemotypes targeting these receptors. Structure-based drug design, now an indispensable component of drug discovery, principally employs methods of receptor-based virtual screening and molecular docking for binding pose prediction [12], hit identification, and lead optimization [13]. As part of our ongoing effort to discover new MOR modulators with novel structures, the study herein described focused on the crystal structure of the MOR inactive-state for the discovery of novel MOR ligands using VS. In particular, we performed a VS study employing an in-house built library of tetrapeptide compounds with the aim to identify novel peptides acting as MOR modulators (Figure 2). To the best of our knowledge, the study herein reported represents one of the first examples of a VS campaign focused on the identification of new peptide ligands as opioid modulators.

## 2. Material and Methods

### 2.1. Peptide Library Generation

The tetrapeptide library has been built using an in-house python program that generates all possible four-letter strings obtained by the combination of the 20 one-letter codes corresponding to the 20 standard amino acids. In this way, the program generated 160,000 different four-letter strings each corresponding to a different tetrapeptide [14]. By including alternative protonation states for some residues (e.g., for histidines), the number of different four-letter strings rose to about 198,000. The obtained 1D library was then converted to a database containing the 3D chemical structures of all the tetrapeptides encoded by the four-letter strings using the Maestro suite [15].

### 2.2. Pharmacophore Modeling

The receptor-based pharmacophore model was generated based on the X-ray structure of the µ-opioid receptor bound to the morphinan antagonist β-funaltrexamine (β-FNA) (PDB code 4DKL) [16]. Prior to pharmacophore generation, the chain of β-FNA connecting the covalent ligand to residue K233 of the protein was removed from the ligand. The complex of MOR bound to the core scaffold of the ligand was then subjected to energy minimization using the software AMBER 14 [17]. The complex was placed in a rectangular parallelepiped water box (TIP3P explicit solvent model) and solvated with a 15 Å water cap. Chlorine ions were added as counterions in order to neutralize the system. General amber force field (GAFF) parameters were provided to the ligand, while partial charges were determined using the AM1-BCC method. Two steps of energy minimization were performed. In the first step, a position constraint of 100 kcal/(mol ·Å^2^) was imposed on the whole complex, so that only water molecules were minimized. In the second one, a harmonic potential of 10 kcal/(mol ·Å^2^) was applied only to the protein α carbons; in this way, the whole system was energy minimized through 10,000 steps of steepest descent followed by conjugate gradient, until a convergence of 0.05 kcal/(mol · Å^2^) was reached. The software LigandScout 4.08 [18] was used to build the receptor-based pharmacophore models starting from the minimized complex. An exhaustive pharmacophore model including all the possible features identified by the program was generated and only the five desired features, representing the ligand-protein interactions established by the core scaffold of β-FNA (see Results and Discussion for details), were then retained in the final model. The H-bond acceptor feature representing the interaction with H297 was manually generated by averaging the coordinates of the hydroxyl group of the ligand forming the water-bridged interaction with H297 and the two structural water molecules participating in the H-bond network. The final pharmacophore model included 5 total features and the excluded volume spheres, which were defined on the basis of the receptor structure as implemented in the default LigandScout configuration.

### 2.3. Database Generation and Pharmacophore Screening

The previously created library of tetrapeptides, including about 198,000 compounds, was used as the screening database. The software Icon [19] implemented in LigandScout was employed to perform ligand conformational sampling and to create the LigandScout 3D database through the idbgen utility of LigandScout. The generated database, which included up to 100 conformers for each tetrapeptide ligand, was then screened using the previously created receptor-based pharmacophore model and imposing the positively charged feature as mandatory. Therefore, only the compounds respecting the volume constraints, matching the positively charged feature, and at least two additional features were retained by the filter. The pharmacophore model and the pharmacophore filtering parameters were validated using the tetrapeptide Tyr–Gly–Gly–Phe (also included in the screening database), corresponding to the common amino-terminal sequence of natural opioid peptides, and the DOR-selective natural peptide product rubiscolin-5 [20]. The pharmacophore filter successfully retained the opioid motif Tyr–Gly–Gly–Phe and discarded rubiscolin-5. The reliability of the pharmacophore filter was thus confirmed.

### 2.4. Docking Reliability Evaluation

The reliability of 10 different docking procedures in predicting the putative binding mode of small peptides has been evaluated through self-docking studies performed using a heterogeneous set of X-ray co-crystal structures of tetrapeptide and pentapeptide ligands in complex with their receptors, available in the Protein Data Bank [21] (PDB IDs 1SUA, 1BHF, 1YTI, 2O1N, 2Z91, 3PA7, 4C2C, 4PRY, 4RWD, 4SGA, 5E6O, 5SGA). Therefore, a total of 12 different X-ray ligand-protein complexes was used for self-docking analyses. Autodock 4.2.3, Dock 6.5, Gold 5.1 with the four implemented fitness functions (i.e., GoldScore, ChemScore, Astex Statistical Potential, and ChemPLP), Autodock Vina 1.1 and Glide 5.0 with the standard-precision (SP), extra-precision (XP) and high-throughput virtual screening (HTVS) methods were employed for this evaluation, as previously described [21,22,23]. For all the 10 procedures, the binding cavity used for the docking calculations was defined in order to include all residues located within 25 Å from the center of the co-crystallized peptide ligand. In the docking studies performed with Gold [24] the “allow early termination” option was deactivated, while the possibility for the ligand to flip ring corners was enabled. The ligands were subjected to 30 genetic algorithm runs and Gold defaults were used for all other settings. Autodock [25] calculations were carried out, performing 20 runs of Lamarckian genetic algorithms for each ligand, with 2,500,000 steps of energy evaluation, while all other settings were left as their defaults. For Autodock Vina [25] calculations, the exhaustiveness parameter was set to 10 and the Energy_range to 1, whereas default values were used for all other parameters. In the docking studies performed using Dock [25] and Glide [26], all settings were left as their defaults. The root-mean-square deviation (RMSD) of the backbone of the peptide ligands in their docking poses with respect to their crystallographic conformation was calculated using the rms_analysis software of the Gold suite.

### 2.5. Docking of Screening Compounds and Pose Filtering

The screening compounds selected through the pharmacophore-based filter were docked into the X-ray structure of the µ-opioid receptor bound to the morphinan antagonist β-FNA (PDB code 4DKL). The same energy minimized protein structure used for pharmacophore modeling was employed for docking studies; however, the two structural water molecules participating in the H-bond network with H297 were removed from the receptor so that the docked ligands could directly interact with H297. Docking calculations for the screened compounds were performed using Glide with SP method and Autodock Vina, using the same settings employed in the reliability analysis.

The filtering of the docking results was carried out by superimposing the docked compounds to the pharmacophore model directly from the supplied poses, without changing their coordinates. In this way, it was possible to select only ligands for which a binding mode matching the required pharmacophore features was predicted by docking, as already performed in previous successful virtual screening studies [27,28,29,30]. The retrieval of compounds matching the positively charged feature of the model and at least two additional features was imposed in this search.

### 2.6. Molecular Dynamics

All simulations were carried out using AMBER 14 [17]. The complexes were placed in a rectangular parallelepiped water box (TIP3P explicit solvent model) and solvated with a 15 Å water cap. Chlorine ions were added as counterions in order to neutralize the system. General amber force field (GAFF) parameters were provided to the ligands, while partial charges were determined using the AM1−BCC method. Prior to running the molecular dynamics (MD) simulations, two steps of energy minimization were performed, as described before. Particle mesh Ewald (PME) electrostatics and periodic boundary conditions were used in the simulation. The minimized structures were employed as starting conformations for the MD simulations. The time step of the simulations was 2 fs, a cutoff of 10 Å was set for the non-bonded interactions and the SHAKE algorithm was employed to keep all bonds involving hydrogen atoms rigid. A constant volume MD simulation was performed for the first 500 ps during which the temperature of the system was increased from 0 to 300 K. Then, the system was equilibrated through 2 ns of constant-pressure simulation, which was carried out maintaining a constant temperature of 300 K by using the Langevin thermostat. Finally, an additional 10 ns of constant-pressure MD simulations were performed. During all MD steps, a position restraint of 10 kcal/(mol · Å^2^) was applied to the protein α carbons.

## 3. Chemistry

### 3.1. Materials and Methods

Solvents and reagents were purchased from WVR (Milano, Italy) and used as supplied without further purification. Amino acids were purchased from GLS Shanghai (Shanghai, China). ^1^H NMR spectra were recorded at 25 °C on a 300 MHz Varian Mercury spectrometer. Chemical shifts are reported in parts per million (*δ*) downfield from the internal standard TMS. Mass spectra were recorded on an LCQ Finnigan-Mat mass spectrometer (San Jose, CA, USA) by ESI-spay source and ion trap analyzer. The capillary temperature was set at 200 °C and the spray voltage at 4.00 kV. The electrospray was formed by using nitrogen (N_2_) as both the sheath gas and the auxiliary gas with a flow of helium. The purity of the final TFA salts was confirmed by NMR analysis, ESI-LRMS, and analytical RP-HPLC recorded at 216, 235, 254, and 275 nm (Waters C18 4.6 mm × 150 mm) at a flow rate of 1 mL/min, using as eluent a gradient of H_2_O/acetonitrile-0.1% TFA ranging from 5% ACN to 90% ACN in 32 min, and was found to be ≥95% (see Supporting Information).

### 3.2. Synthesis of Compounds

**Scheme 1:**
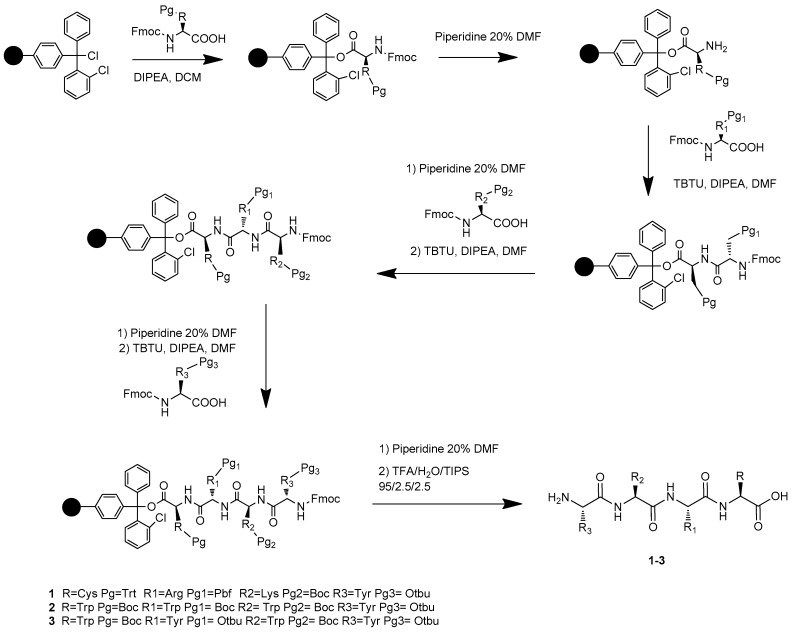
Preparation of products 1–3 via solid-phase peptide synthesis (SPPS).

The synthesis of the novel free *C*-terminal peptides 1, 2, and 3 was performed by solid-phase peptide synthesis (SPPS) [31,32,33] on Cl-Trt chloride resin (0.1 mmol scales; loading: 1.6 mmol Cl-/g). All amino acids have the Fmoc-*N*-terminus and the following side-chain protecting groups: *tert*-butyloxy-carbonyl (Boc) for Lysine and Tryptophan, 2,2,4,6,7-Pentamethyldihydrobenzofuran-5-sulfonyl (Pbf) for Arginine, O-*tert*-butyl (O*t*Bu) for Tyrosine, trityl (Trt) for Cysteine. TBTU/HOBt coupling reagents and DIPEA as a base were used as a coupling mixture, piperidine 20% in DMF for Fmoc group deprotection, as previously reported by our group (Scheme 1) [34,35,36,37]. Kaiser test was applied to check the reaction completeness. All final compounds have been purified in RP-HPLC and were obtained as TFA salts in good yields.

### 3.3. Resin Loading

The 2-Chlorotrityl chloride resin was swelled in DCM. The first Fmoc-protected amino acid (3 equiv) and DIPEA (6 equiv) were dissolved in DCM (5 mL), then the mixture was added to the resin. The coupling was shacked vigorously overnight. After the resin was washed with DMF/DCM and treated with a solution of DCM (17 mL)/MeOH (2 mL)/DIPEA (1 mL) three times for five minutes, washed with DMF/MeOH/DCM and deprotected with a piperidine 20% solution in DMF. The Kaiser test was routinely used to check the coupling reaction. Peptide elongation was performed by dissolving the Fmoc-protected amino acid (3 equiv), HOBt anhydrous (3 equiv), and DIPEA (6 equiv) in 5 mL of DMF, then TBTU (3 equiv) was added. The reaction mixture was stirred at room temperature overnight.

### 3.4. Cleavage and Purification

In order to cleave the peptide from the resin, a mixture of TFA/H_2_O/TIPS 95:2.5:2.5 (8 mL for 1 h) was added to the plastic vessel and shacked for 2.5 h. After this time the solution was collected, concentrated under vacuum and the resulting 1 mL of solution was precipitated in cold ether. The peptide was separated from the solution by centrifugation at 4000 rpm for 5 min and the ether removed. This process was repeated four times and the obtained solid was then dried at reduced pressure. Final peptides 1, 2, and 3 were purified by RP-HPLC using a Waters XBridge prep C18, 5.0 μm, 250 mm × 10 mm column at a flow rate of 7 mL/min on a Waters pump 600, using as eluent a linear gradient of H_2_O + 0.1% TFA/CAN + 0.1% TFA, from 5% ACN to 90% ACN in 32 min.

## 4. Biological Assays

### 4.1. Chemicals

Tris-HCl, EGTA, NaCl, MgCl_2_ × 6H_2_O, GDP, the GTP analogue GTPγS were purchased from Sigma-Aldrich (Budapest, Hungary). The highly selective MOR agonist Tyr-D-Ala-Gly-(NMe)Phe-Gly-ol (DAMGO) was obtained from Bachem Holding AG (Bubendorf, Switzerland), the highly selective DOR agonist Ile^5,6^-deltorphin II (IleDelt II) was synthesized in the Laboratory of Chemical Biology group of the Biological Research Center (BRC, Szeged, Hungary). Test ligands were dissolved in 22% DMSO (DAMGO and IleDelt II in water) and were stored in 1 mM stock solution at −20 °C. The radiolabeled GTP analog, [^35^S]GTPγS (specific activity: 1000 Ci/mmol) was purchased from Hartmann Analytic (Braunschweig, Germany). [^3^H]DAMGO (specific activity: 38.8 Ci/mmol), [^3^H]IleDelt II (specific activity: 19,6 Ci/mmol) [38] were radiolabeled by the Laboratory of Chemical Biology group in BRC (Szeged, Hungary).

### 4.2. Animals

For membrane homogenate preparations male Wistar rats (250–300 g body weight) were used. Rats were housed in the local animal house of BRC (Szeged, Hungary). Animals were kept in a temperature-controlled room (21–24 °C) under a 12:12 light and dark cycle and were provided with water and food ad libitum. All housing and experiments were conducted in accordance with the European Communities Council Directives (2010/63/EU) and the Hungarian Act for the Protection of Animals in Research (XXVIII.tv. 32.§). The total number of animals, as well as their suffering, was minimized.

### 4.3. Rat Membrane Preparations

Rats were decapitated and their brains were quickly removed. The brains were used for membrane preparation according to Benyhe et al. [39] partly used for competition binding experiments and partly were further prepared for the [^35^S]GTPγS binding experiments based on Zádor et al. [40]. Briefly, the brains were homogenized, centrifuged in ice-cold 50 mM Tris-HCl (pH 7.4) buffer and incubated at 37 °C for 30 min in a shaking water-bath. After incubation, the centrifugation was repeated as described before and the final pellet was suspended in 50 mM Tris-HCl pH 7.4 buffer containing 0.32 M sucrose and stored at −80 °C. For the [^35^S]GTPγS binding experiments the final pellet of rat brain membrane homogenate was suspended in ice-cold TEM (Tris-HCl, EGTA, MgCl_2_) and stored at −80 °C for further use.

### 4.4. Radioligand Competition Binding Experiments

The test compounds were incubated in 3 and 10 µM concentrations together with rat brain membranes containing 0.3–0.5 mg/mL of protein with ~1 nM [^3^H]DAMGO or [^3^H]IleDelt II in 35 °C for 45 min. Additionally, unlabeled DAMGO and IleDelt II were also incubated together with their labeled counterparts in 3 and 10 µM concentrations for control. Total and non-specific binding was determined in the absence of ligands and the presence of 10 µM unlabeled naloxone, respectively. Following incubation, the bound and unbound radioligands were separated by rapid vacuum filtration through Whatman GF/C glass fibers and washed three times with 5 mL ice-cold 50 mM Tris-HCl (pH: 7.4). The radioactivity of the filters was detected in UltimaGold^TM^ MV aqueous scintillation cocktail with Packard Tricarb 2300TR liquid scintillation counter. The competition binding assays were performed in duplicate and repeated at least three times.

### 4.5. Functional [^35^S]GTPγS Binding Experiments

In [^35^S]GTPγS binding experiments was measured the GDP→GTP exchange of the G_αi/o_ protein in the presence of a given ligand to measure ligand potency and the maximal efficacy of receptors G-protein. The nucleotide exchange is monitored by a radioactive, non-hydrolysable GTP analog, [^35^S]GTPγS. The functional [^35^S]GTPγS binding experiments were performed as previously described [41], with some modifications. The test compounds in 10 µM were incubated together with rat brain membrane homogenates containing ~10 μg/mL protein at 30 °C for 60 min in Tris-EGTA buffer (pH 7.4) composed of 50 mM Tris-HCl, 1 mM EGTA, 3 mM MgCl_2_, 100 mM NaCl, containing 20 MBq/0.05 cm^3^ [^35^S]GTPγS (0.05 nM). Additionally, peptide 1 was also co-incubated with 10 µM DAMGO and cyprodime. Total binding was measured in the absence of test compounds, while non-specific binding was determined in the presence of 10 μM unlabeled GTPγS. The bound and unbound [^35^S]GTPγS were separated as described in the competition binding assays section through Whatmann GF/B glass fibers. The radioactivity of the filters was also detected as described above. [^35^S]GTPγS binding experiments were performed in triplicates and repeated at least three times.

### 4.6. Data Analysis

The specific binding of all radiolabeled compounds was calculated by the subtraction of non-specific binding from total binding and was given in percentage. The data were normalized to total specific binding, which was settled 100%, which in the case of [^35^S]GTPγS also represents the basal activity of the G-protein. Experimental data were presented as mean ± S.E.M. Statistical analyses were performed with GraphPad Prism 5.0 applying one-way ANOVA with Tukey’s multiple comparison test. Significance was accepted at the *p* < 0.05 level.

## 5. Results and Discussion

**Figure 3:**
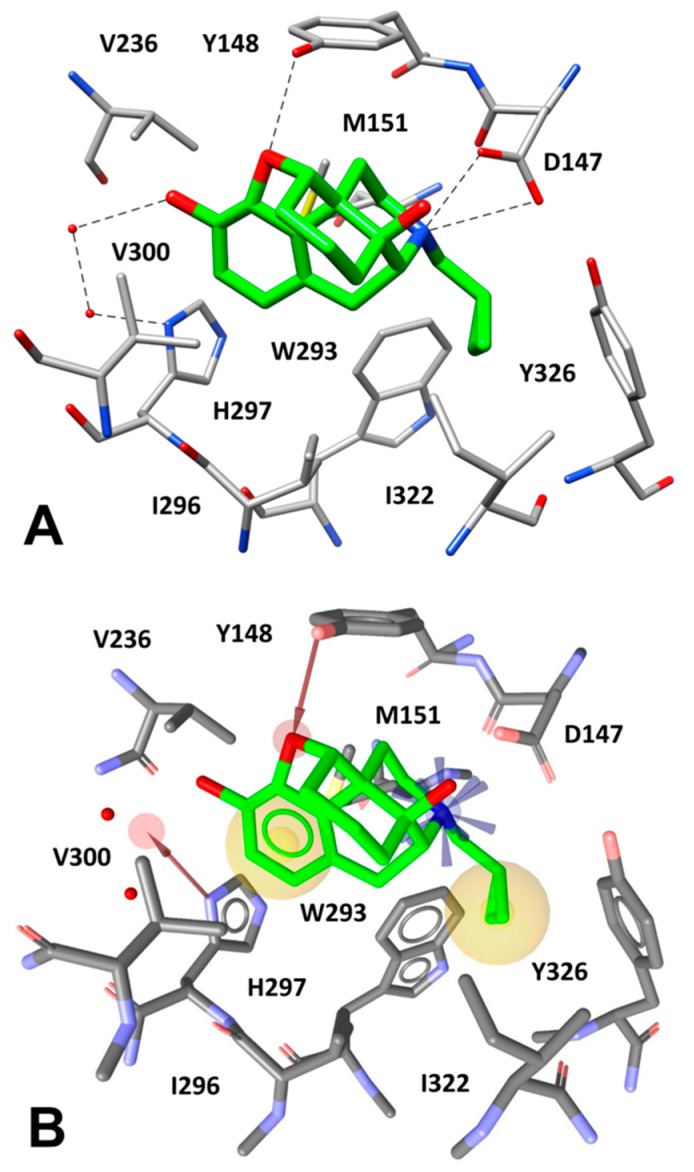
(**A**) Interactions of β-FNA core scaffold (green) inside MOR binding site (gray) and (**B**) receptor-based pharmacophore model used for the VS study, superimposed to the complex.

The µ-opioid receptor (MOR) is the main mediator of narcotic analgesia and addiction, exhibiting a basal signaling activity in SH-SY5Y cells and transfected HEK293 cells via Gαi/Gαo proteins [42]. With the aim to identify novel tetrapeptides able to bind MOR, we performed a VS study using a virtual combinatorial library of tetrapeptides. By using an in-house python program, we generated the FASTA sequences of all the possible tetrapeptides obtained by the combination of the 20 standard amino acids, which were then converted to the corresponding 3D structures (see Material and Methods for details). In this way, we obtained a virtual library of about 200,000 tetrapeptides to be used for VS studies. A receptor-based pharmacophore model was then developed on the basis of the X-ray structure of MOR bound to the morphinan antagonist β-funaltrexamine (β-FNA) [16]. The ligand co-crystallized with MOR is an irreversible antagonist that covalently binds to a lysine residue of the receptor (K233); however, since we aimed at identifying novel peptides with MOR inhibitory activity (i.e., acting as antagonists/inverse agonists), the choice of this X-ray structure, which is the only crystal structure of MOR in inactive conformation, was necessary for our VS protocol. Nevertheless, the pharmacophore model was built taking into account the ligand-protein interactions established by the core scaffold of the antagonist. As shown in Figure 3A, the morphinan core of the ligand forms a salt bridge interaction with D147 through its tertiary amine group, while an H-bond between Y148 and the endocyclic oxygen of the ligand is observed. Moreover, the inhibitor forms a water-bridged interaction with H297 allowed by two structural water molecules that participate in an H-bond network between the side chain of H297 and the phenolic OH group of the ligand. Finally, multiple hydrophobic interactions can be observed between the morphinan core of the inhibitor and the surrounding protein residues. In particular, the ligand cyclopropyl group is placed in a lipophilic pocket delimited by W293, I296, I322, and Y326, thus forming hydrophobic interactions with these residues, while the aromatic ring is sandwiched between M151 and V300, taking additional lipophilic contacts with I296 and H297. Based on these considerations, a receptor-based pharmacophore model comprising five different features was generated. The model included: a) a positively charged feature representing the salt bridge interaction with D147, b) two hydrophobic features representing the lipophilic interactions formed by the cyclopropyl group and the aromatic ring of the ligand, c) two H-bond acceptor features representing the direct and the water-mediated H-bond interactions formed with Y148 and H297, respectively (Figure 3B). It is worth specifying that this last interaction was modeled through a feature pointing at the region of space among the ligand OH group and the two water molecules involved in the H-bond network, in order to find ligands that could replace the structural waters and directly interact with H297. In addition, the model was refined by adding excluded volume spheres mimicking the steric hindrance represented by the MOR binding site (see Materials and Methods for details).

The receptor-based pharmacophore model was used to screen the virtual library of about 200,000 tetrapeptides in order to identify all peptides able to form the key interactions with MOR. For this purpose, only the tetrapeptides respecting the volume constrains, matching the positively charged feature of the model and at least two additional features were retained by the filter. By using this strategy, only 28,070 peptide ligands were selected and subjected to further analyses based on docking evaluations. Interestingly, we found that 5170 out of the 28,070 peptides retrieved by the pharmacophore filter showed a tyrosine residue in the first position. Therefore, as expected, the filter enriched in peptides containing an amino-terminal tyrosine (as in the natural opioid motif Tyr–Gly–Gly–Phe), the subset of compounds to be considered in the docking step.

**Figure 4:**
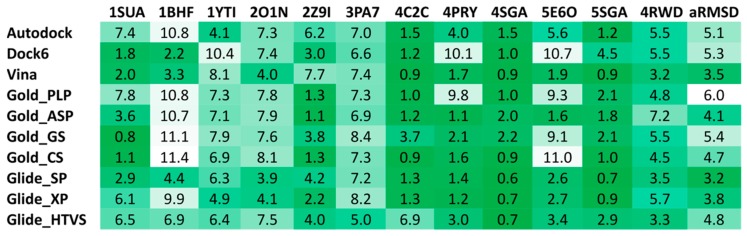
Results of the docking reliability analysis. The root-mean square deviation (RMSD) results obtained for the self-docking studies are shown in the color scale from green (low values) to white (high values).

To the best of our knowledge, no examples of VS studies focused on peptide libraries have been reported to date; as a consequence, no hints about the reliability of the commonly used docking software in predicting the binding mode of small peptides were available in the literature. For this reason, we performed a docking reliability analysis aimed at assessing the performance of 10 different docking procedures in reproducing the experimental binding mode of 12 small peptides for which ligand-protein co-crystal structures were available in the Protein Data Bank. For each peptide docked into its corresponding receptor using the 10 different docking procedures, the root-mean square deviation (RMSD) of the peptide backbone calculated between the binding modes predicted by docking and the corresponding experimental pose were used to evaluate the reliability of the docking methods. As reported in Figure 4, Glide with the standard precision (SP) method and Autodock Vina showed the best results in terms of average RMSD (aRMSD) obtained for the whole dataset of peptides, with values below or equal to 3.5 Å. Although it would have been desirable to obtain RMSD values closer to 2.0 Å, these results were expected, since the docking software was developed and calibrated for the docking of small-molecules with non-peptide structures. Nevertheless, since Glide SP and Autodock Vina performed better, on average, than all other software and thus represented the best docking methods among those tested for the docking of small peptides, these two procedures were selected to be applied in our VS study.

**Figure 5:**
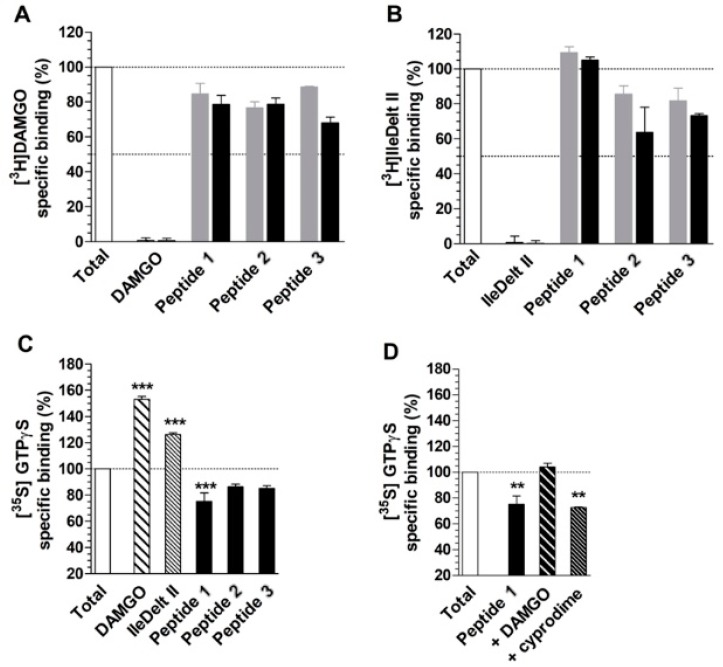
Competition binding (A and B) and G-protein activity (C and D) of peptides 1, 2 and 3. (**A**) and (**B)** represent the specific binding of the indicated radioligands as a percentage, in presence of 3 (grey colored) and 10 µM (black colored) of the indicated ligands. For control, the unlabeled homologous ligands of the radioligands are also indicated. (**C**) and (**D**) represent the specific binding of [^35^S]GTPγS as a percentage (mean ± S.E.M.), in presence of the indicated ligands at 10 µM concentrations. (**D**) MOR specificity of peptide **1** in G-protein activity by displaying the [^35^S]GTPγS specific binding (mean ± S.E.M.) in the absence and presence of 10 µM concentrations of DAMGO and cyprodime, MOR selective agonist and antagonist, respectively. In all cases “Total” represents the specific binding of the radioligand or [^35^S]GTPγS in the absence of the ligands. In the case of [^35^S]GTPγS, it also represents the basal activity of the monitored G-protein. The dotted lines indicate the level of total binding (100%) and 50% radioligand specific binding inhibition (**A** and **B**) or G-protein basal activity (**C** and **D**). ***: *p* > 0.001; **: *p* > 0.01. The experiments and data analysis were performed as described under section “[^35^S]GTPγS binding experiments” and “Data Analysis” within the “Materials and Methods”

The 28,070 peptide ligands previously selected through the pharmacophore screening, were thus initially docked into MOR binding site, by using Glide SP and ranked according to the docking score associated to their predicted binding mode. Since in our previous studies Glide SP scoring function showed to be particularly reliable [43,44], and all peptides accurately docked by Glide SP in the reliability analysis showed docking scores lower than −8.0 kcal/mol, all screening compounds for which a docking score higher than –8.0 kcal/mol was obtained were discarded, while the remaining top-scored 913 peptides were subjected to a qualitative post-docking filter. Precisely, the selected compounds were superimposed with the receptor-based pharmacophore model and only those still matching the positively charged feature and at least two additional features of the model in their predicted binding mode were further considered. Based on this qualitative filter, only 146 tertrapeptides were retained and subjected to additional docking studies using Autodock Vina. The docked compounds were directly subjected to the pharmacophore-based post-docking filter and only 15 tetrapetides showed to maintain the ligand-protein interactions represented by the model. These compounds were thus further analyzed through molecular dynamics (MD) simulations studies, in order to evaluate the stability of their binding disposition into MOR. A total of 12.5 ns of MD simulations with explicit solvent were performed for each ligand and the persistence of the key pharmacophoric interactions during the simulations was analyzed. The peptide ligands that couldn’t maintain the salt bridge interaction with D147 and at least one of the H-bonds with H297 and Y148 for at least 80% of the whole MD simulation were discarded. As a result, only 3 tetrapeptides were considered as potential MOR ligands and were then synthesized to be tested for their biological activity: peptide 1 (Tyr–Lys–Arg–Cys), peptide 2 (Tyr–Trp–Trp–Trp) and peptide 3 (Tyr–Trp–Tyr-Trp). Interestingly, the three selected compounds presented a tyrosine residue in the first position as observed in the natural opioid motif (Tyr–Gly–Gly–Phe). For initial screening purposes, the compounds were tested in two high concentration points, 3 and 10 µM. However, the peptides did not show a strong affinity for MOR nor for DOR, since none of the test compounds inhibited radioligand total specific binding lower than 50% even at the highest (10 µM) concentrations (Figure 5A and 5B). For comparison, DAMGO and IleDelt II, MOR and DOR selective agonist compounds, respectively, inhibited the specific binding of their radiolabeled homologs to non-specific binding level (0%) (Figure 5A,B).

The G-protein activity of the test compounds was firstly investigated at 10 µM concentrations. The results were in agreement with the affinity data, with the only exception of peptide 1, since peptide 2 and 3 did not alter significantly the basal activity (100%) of the monitored G-protein in 10 µM concentrations (Figure 5C). For comparison, DAMGO and IleDelt II selective ligands for MOR and DOR respectively, significantly increased the specific binding of the [^35^S]GTPγS compared to the basal level, which demonstrates their well-documented agonist activity. However, peptide 1 displayed an appreciable inverse agonist effect, indicated by a reduced (~20%) G-protein basal activity (Figure 5C). Since in the binding affinity assay peptide 1 displayed inhibition of [^3^H]DAMGO specific binding and not against [^3^H]IleDelt II, we investigated the MOR specificity of the inverse agonist effect. When DAMGO was added to peptide 1 at 10 µM concentration, the inverse agonist effect was reversed back to basal activity level, indicating a MOR mediated effect. Interestingly the MOR selective antagonist cyprodime did not cause any significant change (Figure 5D), indicating that our receptor-based VS protocol yielded the discovery of a novel peptide acting as an inverse agonist at the MOR receptor.

**Figure 6:**
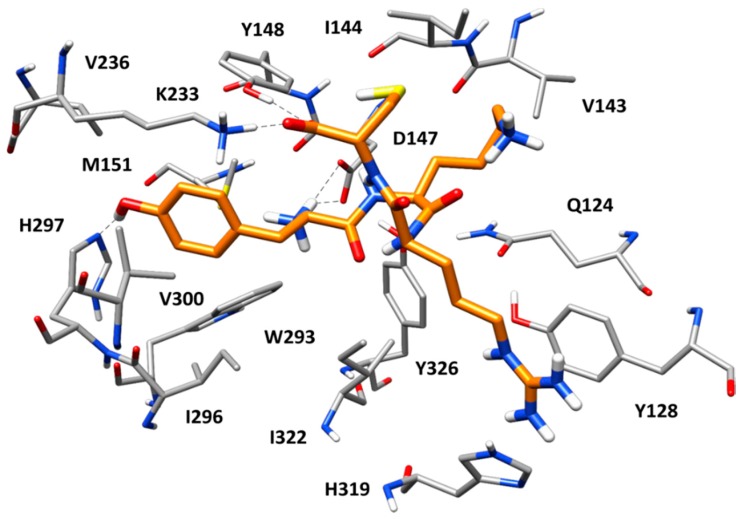
Minimized average structure of peptide 1 (orange) in complex with MOR obtained from the molecular dynamics (MD) simulation. The main ligand-protein H-bond interactions are shown as black dashed lines.

Figure 6 shows the predicted binding mode of peptide 1 within MOR binding site refined through MD simulations. As observed in many endogenous opioid ligands, the tyrosine residue of the peptide forms key interactions with the protein and efficiently mimics the morphinan scaffold of the crystallographic antagonist β-FNA. Precisely, the protonated amino group of the ligand’s tyrosine forms a stable salt bridge with the side chain of D147, which is maintained for the whole MD simulation; the aromatic ring of the residue is placed between M151 and V300, forming hydrophobic interactions with these residues as well as with W293, I296, and H297; finally, the phenolic group of the ligand’s tyrosine succeeds in establishing a direct H-bond with H297, thus replacing the two structural water molecules observed in the reference X-ray structure of MOR. Interestingly, at the beginning of the MD simulation, the imidazole ring of H297 undergoes a 180 degrees rotation, which allows the residue to form an H-bond with the backbone oxygen of W293. Nevertheless, the H-bond between H297 and the ligand is maintained for almost the whole simulation. The cysteine residue of the ligand forms additional interactions with the protein. In particular, the terminal carboxylic group of the peptide showed a stable ionic interaction with the side chain of K223 (the alkylated residue in the reference X-ray complex) and an H-bond with the phenol group of Y148. Finally, the lysine residue of the peptide did not show particularly relevant interactions with the receptor, while the guanidine moiety of the ligand’s arginine residue formed strong a π–π stacking with H319 and Y128. Interestingly, peptides 2 and 3, which resulted to be inactive against MOR, were able to form stable H-bond interactions with both H297 and Y148, as well as the salt bridge with D147, in their predicted binding mode. Moreover, both peptides formed an additional H-bond with W318 not shown by peptide 1 (Figure S1). However, the inactive peptides lacked the stable salt bridge with K233 and the strong π–π stacking with H319 and Y128 shown by peptide 1. Moreover, peptides 2 and 3 were less buried within the protein binding site and more protruded toward the extracellular side, with a widely solvent-exposed tryptophan residue. These binding mode differences with respect to peptide 1 might explain the inactivity of peptides 2 and 3.

## 6. Conclusions

To the best of our knowledge, the study herein reported represents one of the first examples of a VS campaign focused on the identification of new peptide ligands targeting the μ-opioid receptor. In particular, our VS protocol aimed at the discovery of new tetrapeptide ligands with inverse agonist activity at the µ-opioid receptor. For this purpose, an in-house built virtual library of about 200,000 tetrapeptides was generated and filtered using receptor-based pharmacophore screening in combination with docking studies. The top-scored compounds were then analyzed through MD simulation studies and the three most promising peptide ligands were synthesized and subjected to biological evaluation. The most interesting hit, peptide 1, showed selectivity for MOR over DOR and demonstrated an appreciable inverse agonist effect at MOR. In summary, peptide 1 may represent a promising new hit compound to be used as a starting point for structure-based ligand optimization aimed at discovering potent opioid modulators. The use of opioid modulators, such as MOR inverse agonists, might counteract the typical side effects produced by potent analgesic compounds acting as agonists. Moreover, MOR inverse agonists might avoid the adverse effects of naloxone and naltrexone in the treatment of heroin addiction. Therefore, these compounds could become useful in treating narcotic overdose or in the management of drug addiction. Finally, the identification of peptide 1 demonstrates the reliability of the VS protocol herein developed, which could be further applied for the discovery of novel peptide ligands of opioid receptors.

## Supplementary Materials

The following are available online, Synthetic procedures, compounds characterization and analytical data.
