# Supplementary material for: Discovery of Novel µ-Opioid Receptor Inverse Agonist from a Combinatorial Library of Tetrapeptides through Structure-Based Virtual Screening
Source: Molecules. 2019 Oct 27;24(21):3872. doi: 10.3390/molecules24213872 (PMC6865014; doi:10.3390/molecules24213872)

Discovery of novel μ-opioid receptor inverse agonist from a combinatorial library of tetrapeptides through structure-based virtual screening

Giulio Poli,[a] Marilisa Pia Dimmito,[b] Adriano Mollica,[b,*] Gokhan Zengin,[c] Sandor Benyhe,[d] Ferenc Zador,[d] Azzurra Stefanucci[b]

[a] Department of Pharmacy, University of Pisa, Via Bonanno 6, 56126 Pisa, Italy.

[b] Department of Pharmacy, University of Chieti-Pescara "G. d'Annunzio", Via dei Vestini 31, 66100 Chieti, Italy.

[c] Department of Biology, Science Faculty, Selcuk University, Konya, Turkey.

[d] Institute of Biochemistry, Biological Research Center, Hungarian Academy of Sciences, H-6726 Szeged, Temesvári krt. 62., Hungary.

*corresponding author email: a.mollica@unich.it

TABLE OF CONTENTS	PAGES
Compounds characterization	S2
Analytical RP-HPLC traces of peptides **1**, **2** and **3**	S3
LRMS for peptides **1**, **2** and **3**	S4-S6
^{1}H-NMR for peptides **1**, **2** and **3**	S7-S9

COMPOUNDS CHARACTERIZATION:

Peptide 1 (TFA·H-Tyr-Lys-Arg-Cys-OH): white solid powder, 36% yield; rt (analytical RP-HPLC): 12.86 (min). ^{1}H NMR (300 MHz, DMSO-d$_6$) δ 8.63 (t, 1H, NH Arg side chain), 8.37 (d, 1H, NH Arg), 8.24 (t, 2H, NH$_2$ Lys), 8.17 (d, 1H, NH Lys), 7.99 (brs, 3H, NH_3^+ Tyr), 7.75-7.60 (m, 4H, NH guanidinium group), 7.03 (d, 3H, Tyr aromatics + NH Cys), 6.68 (dd, 2H, Tyr aromatics), 4.49-4.30 (m, 2H, CH^{α} Tyr, CH^{α} Arg), 3.96 (m, 1H, CH^{α} Lys), 3.18-2.87 (m, 4H, CH_2^{β} Tyr, CH_2^{β} Cys), 2.76-2.70 (m, 4H, CH_2^{β} Arg, CH_2^{β} Lys), 1.68-1.52 (m, 8H, CH_2^{γ} Arg, CH_2^{δ} Arg, CH_2^{γ} Lys, CH_2^{δ} Lys), 1.31 (m, 2H, CH_2^{ε} Lys). LRMS (ESI): m/z calc. for $C_{24}H_{40}N_8O_6S$ 568.2; found 568.5 [M].

Peptide 2 (TFA·H-Tyr-Trp-Trp-Trp-OH): white solid powder, 42% yield; rt (analytical RP-HPLC): 15.98 (min). ^{1}H NMR (300 MHz, DMSO-d$_6$) δ 10.81, 10.75, 10.74 (m, 3H, NH indole), 8.64 (d, 1H, NH Trp) 8.21 (t, 2H, Trp aromatics), 7.85 (brs, 3H, NH_3^+ Tyr), 7.63-7.49 (td, 3H, 2H' indole), 7.31-7.26 (m, 3H, 1H Trp aromatic + 2 NH Trp), 7.12-6.91 (m, 12H, Trp aromatics + Tyr aromatics + NH Trp), 6.65 (dd, 2H, Tyr aromatics), 4.64-4.49 (m, 3H, CH^{α} Tyr + 2 CH^{α} Trp), 3.57 (m, 1H, CH^{α} Trp under water), 3.18-2.68 (m, 8H, CH_2^{β} Tyr + 3*CH_2^{β} Trp). LRMS (ESI): m/z calc. for $C_{42}H_{41}N_7O_6$ 739.3; found 740.3 $[M+H]^+$.

Peptide 3 (TFA·H-Tyr-Trp-Tyr-Trp-OH): white solid powder, 45% yield; rt (analytical RP-HPLC): 15.40 (min). ^{1}H NMR (300 MHz, DMSO-d$_6$) δ 10.81, 10.76 (d, 2H, NH indole), 8.63 (d, 1H, NH Trp), 8.25 (d, 1H, NH Tyr), 8.13 (d, 1H, NH Trp), 7.86 (brs, 3H, NH_3^+ Tyr), 7.62 (d, 1H, Trp aromatic), 7.54 (d, 1H, Trp aromatic), 7.32-7.27 (m, 2H, Trp aromatics), 7.15-6.89 (m, 10H, 4H Tyr aromatics + 6H Trp aromatics), 6.66 (dd, 2H, Tyr aromatics), 6.55 (dd, 2H, Tyr aromatics) 4.61-4.48 (m, 3H, 2*CH^{α} Tyr + CH^{α} Trp), 3.69 (m, 1H, CH^{α} Trp under water), 3.14-2.67 (m, 8H, CH_2^{β} Trp + CH_2^{β} Tyr). LRMS (ESI): m/z calc. for $C_{40}H_{40}N_6O_7$ 716.2; found 717.3 $[M+H]^+$.

ANALYTICAL RP-HPLC TRACES OF PEPTIDES 1-3

Peptide 1

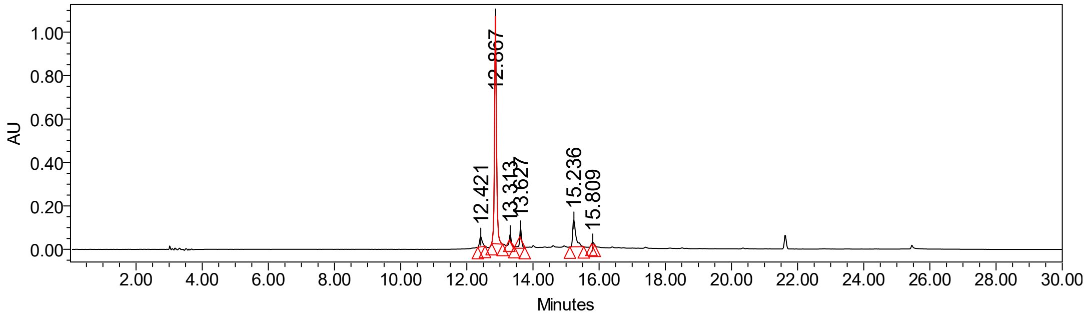

Retention Time	% Area	Height
12.867	95.61	997891
13.313	0.83	31481
13.627	1.61	41667
15.236	4.09	122121
15.809	0.33	9664

Peptide 2

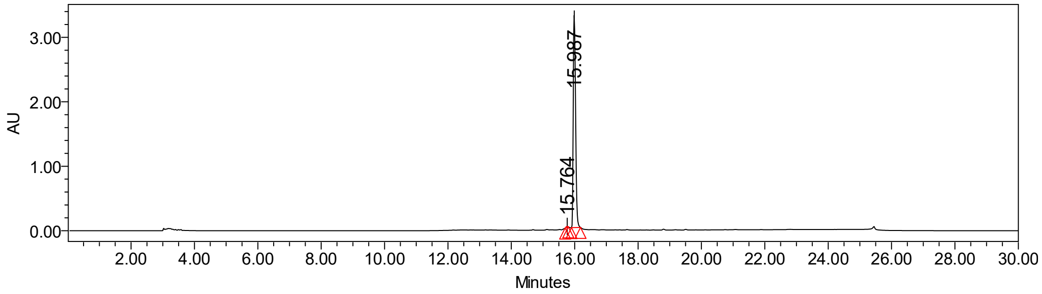

Retention Time	% Area	Height
15.764	0.35	16139
15.987	99.65	3321654

Peptide 3

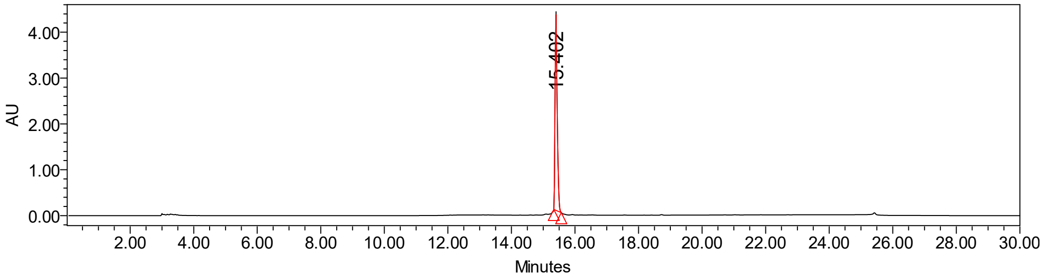

	Name	Retention Time	% Area	Height
1		15.402	100.00	4029300

LRMS for peptides 1-3

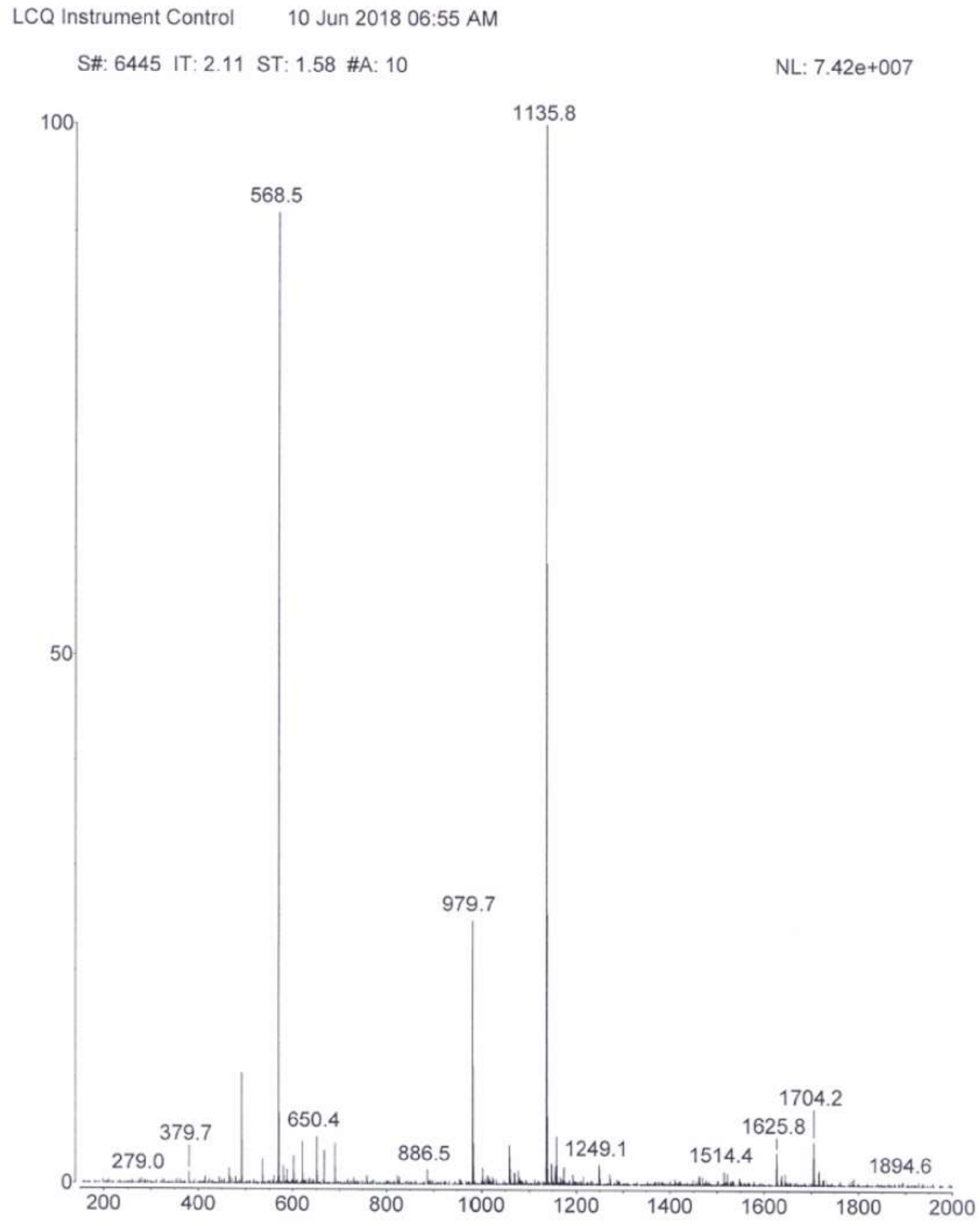

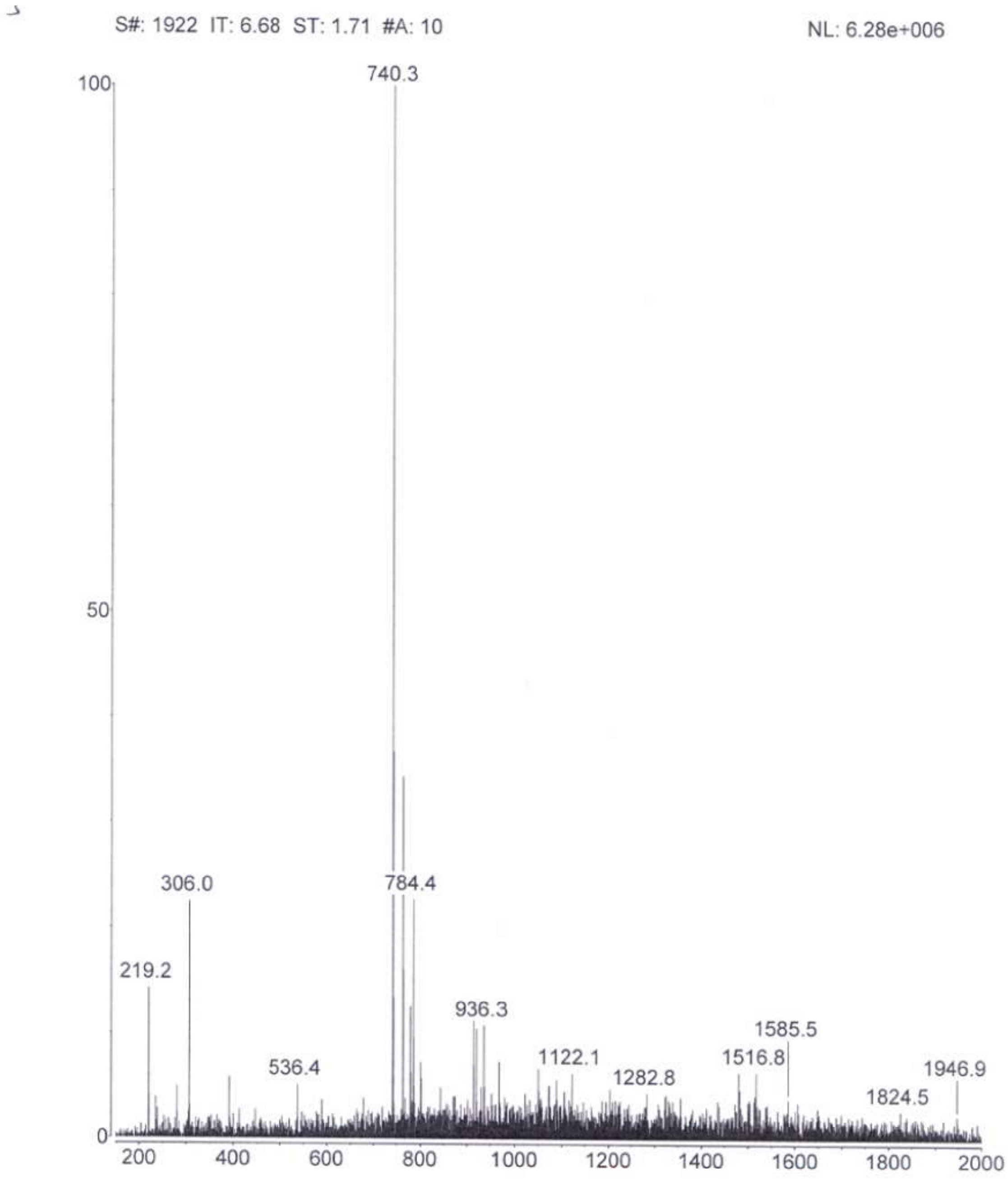
S#: 1922 IT: 6.68 ST: 1.71 #A: 10
NL: 6.28e+006
100
50
0
740.3
306.0
784.4
219.2
936.3
1585.5
536.4
1122.1
1516.8
1946.9
1282.8
1824.5
200
400
600
800
1000
1200
1400
1600
1800
2000

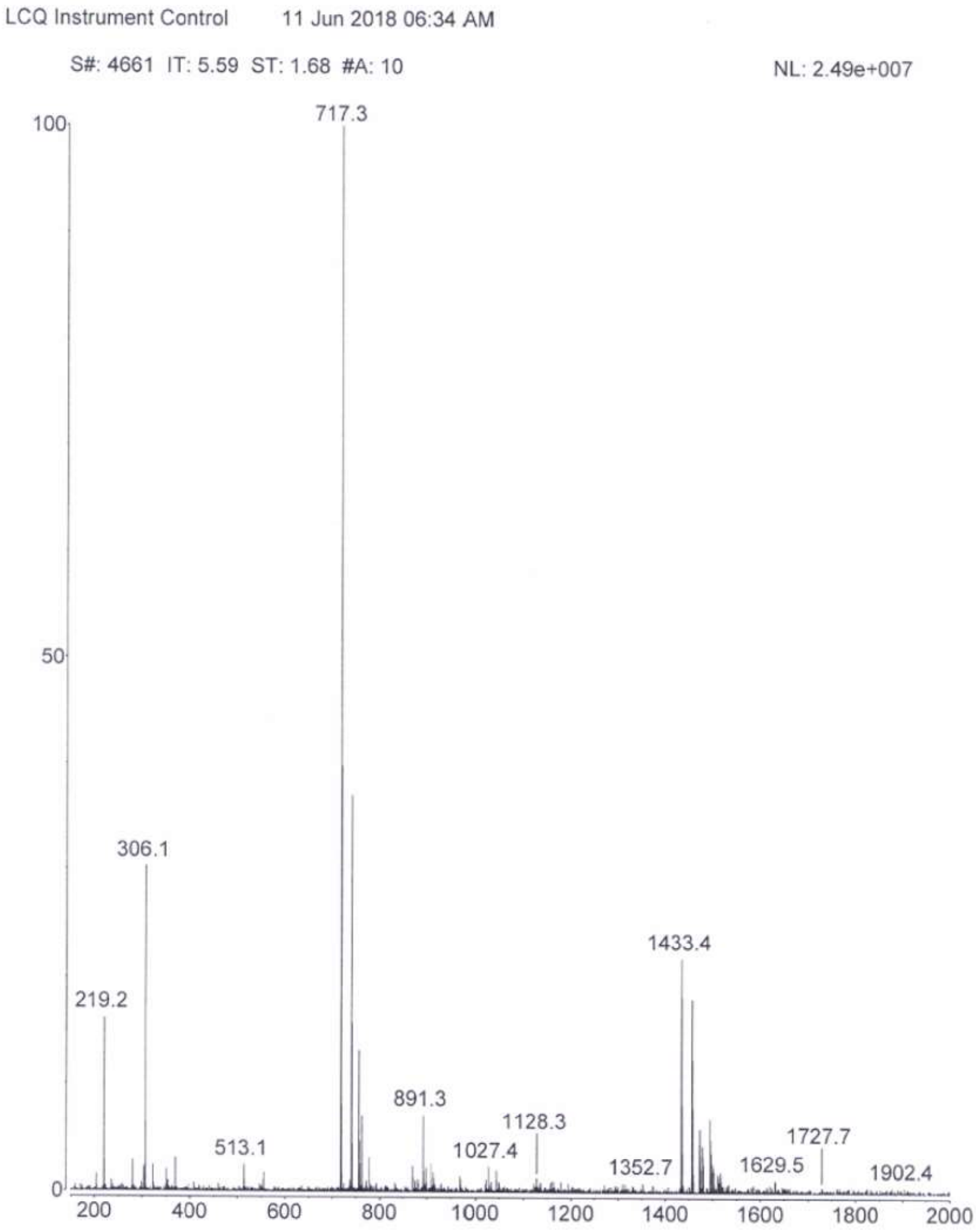

LCQ Instrument Control 11 Jun 2018 06:34 AM
S#: 4661 IT: 5.59 ST: 1.68 #A: 10
NL: 2.49e+007
717.3
306.1
219.2
513.1
891.3
1027.4
1128.3
1352.7
1433.4
1629.5
1727.7
1902.4
100
50
0
200
400
600
800
1000
1200
1400
1600
1800
2000

^{1}H-NMR in DMSO-d_6 for peptide **1**

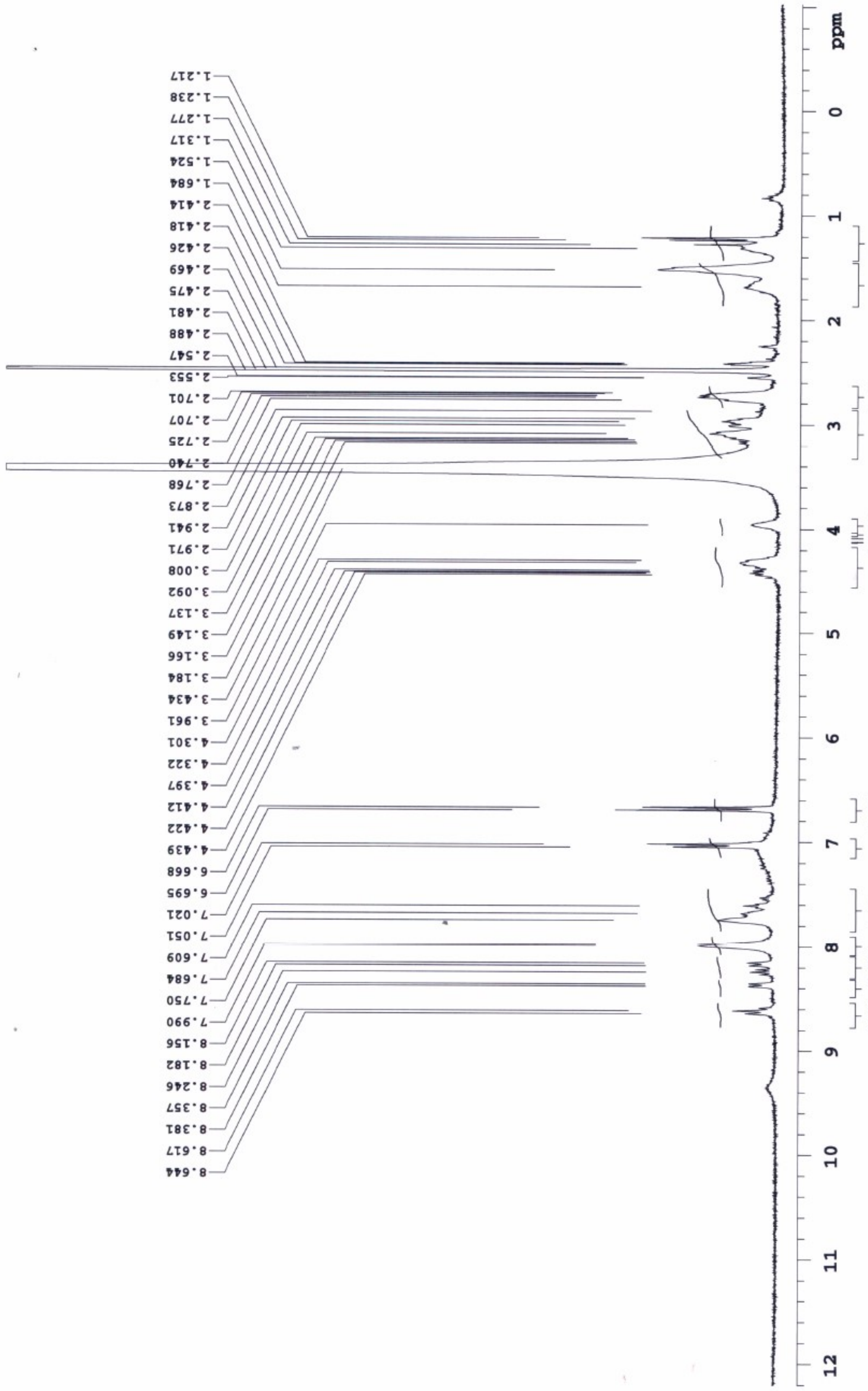

^{1}H-NMR in DMSO-d_6 for peptide **2**

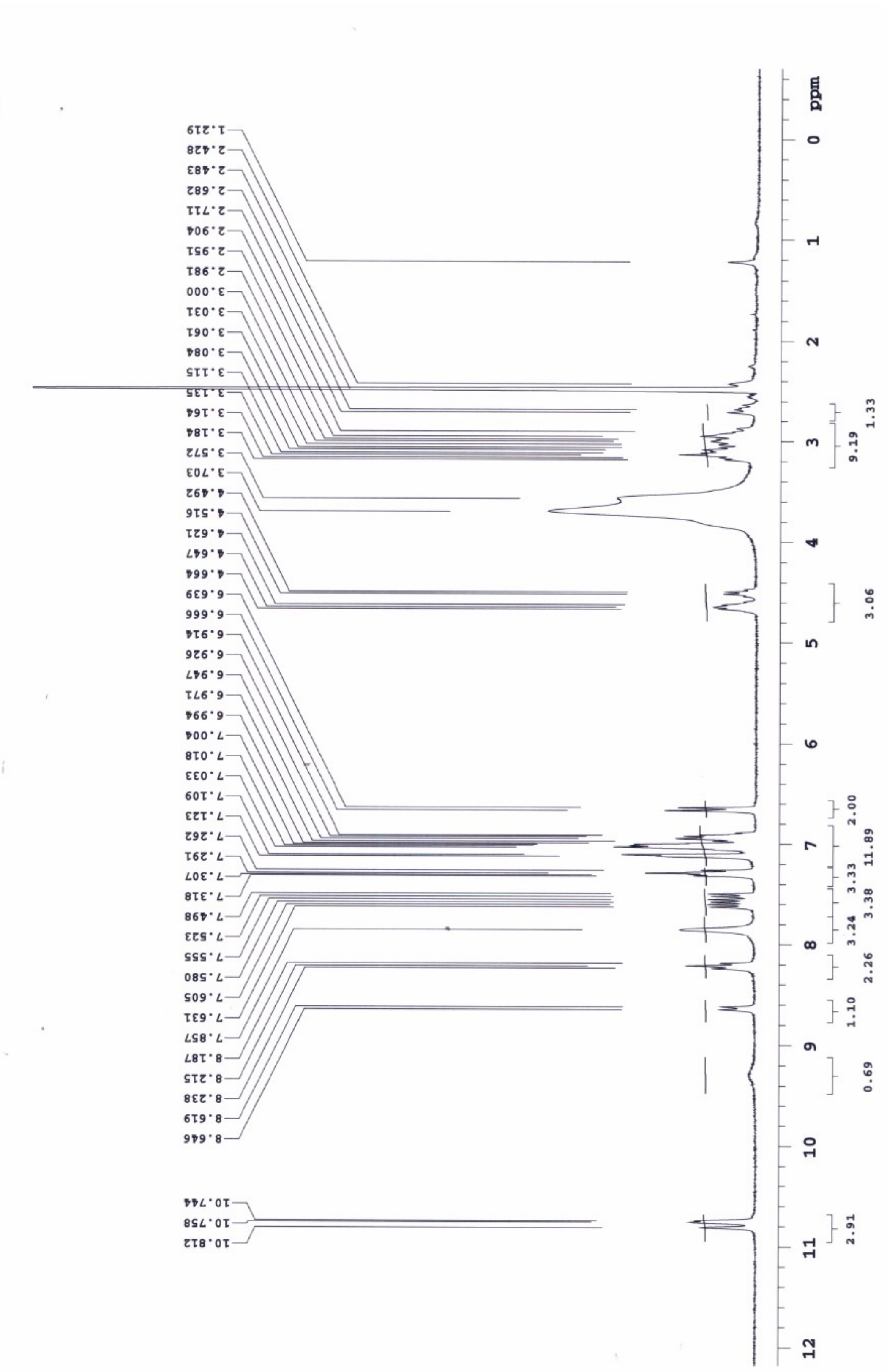

^{1}H-NMR in DMSO-d_6 for peptide **3**

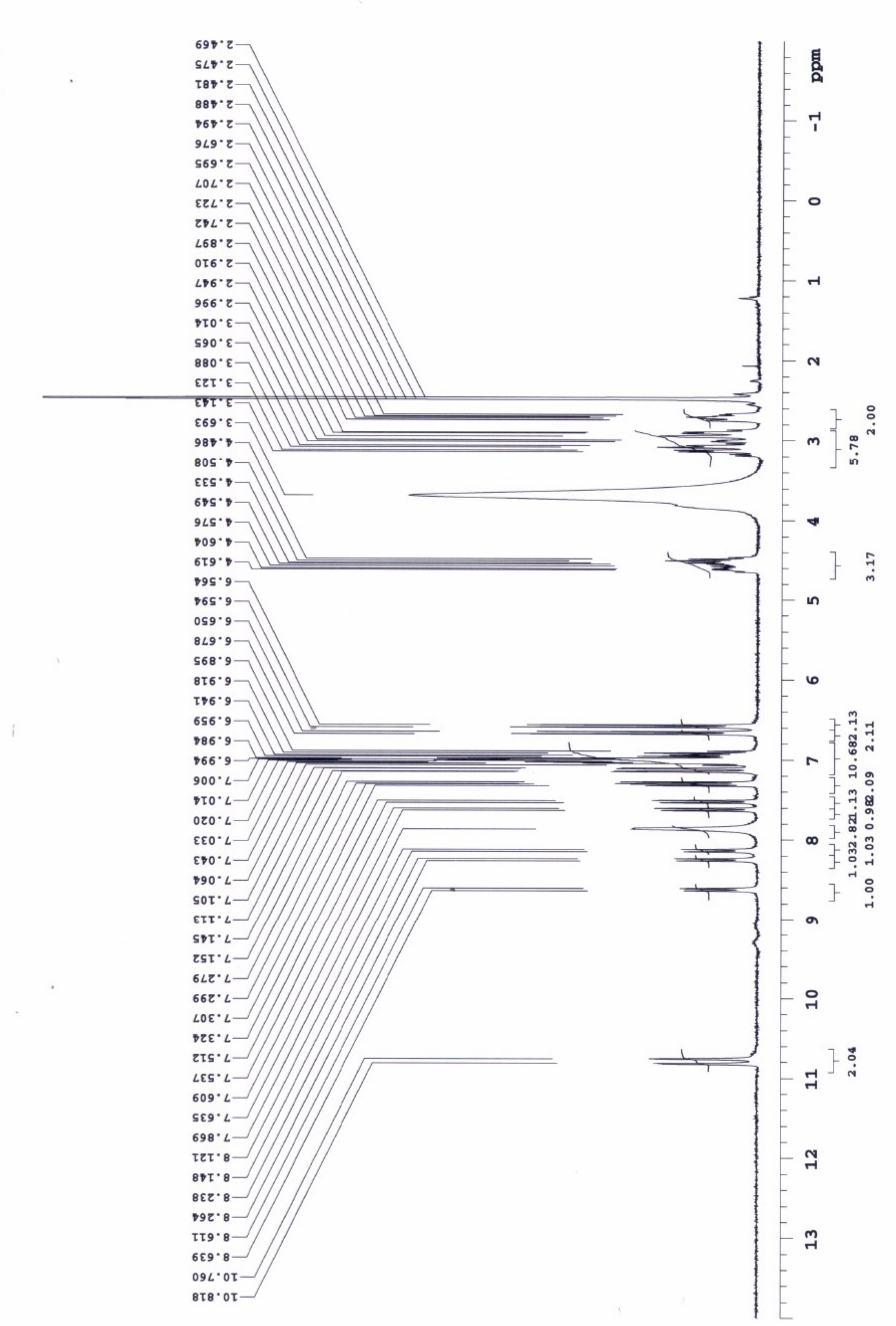

Supplement: Supplementary file 1 [file molecules-24-03872-s001.pdf]
